# Partial loss of CovS function in *Streptococcus pyogenes* causes severe invasive disease

**DOI:** 10.1186/1756-0500-6-126

**Published:** 2013-03-28

**Authors:** Ichiro Tatsuno, Ryo Okada, Yan Zhang, Masanori Isaka, Tadao Hasegawa

**Affiliations:** 1 Department of Bacteriology, Nagoya City University Graduate School of Medical Sciences, 1 Kawasumi Mizuho-cho Mizuho-ku, Nagoya 467-8601, Japan

## Abstract

**Background:**

CovRS (or CsrRS) is a two-component regulatory system that regulates the production of multiple virulence factors in *Streptococcus pyogenes*. *covS* mutations are often found in isolates recovered from mice that have been experimentally infected with *S. pyogenes* and *covS* mutations enhance bacterial virulence in an invasive infection mouse model. In addition, *covS* mutations were detected more frequently in a panel of clinical isolates from severe invasive streptococcal infections than those from non-severe infections. Thus, *covS* mutations may be associated with the onset of severe invasive infections.

**Results:**

Known *covS* mutations were divided into two groups: (i) frameshift mutations that caused a deletion of functional regions and (ii) point mutations that caused single (or double) amino acid(s) substitutions. Frameshift mutations are frequent in mouse-passaged isolates, whereas point mutations are frequent in clinical isolates. The functions of CovS proteins with a single amino acid substitution in clinical isolates were estimated based on the streptococcal pyrogenic exotoxin B (SpeB) production and NAD^+^-glycohydrolase (NADase) activity, which are known to be regulated by the CovRS system. Point mutations partially, but not completely, impaired the function of the *covS* alleles. We also investigated some of the benefits that a partial loss of function in *covS* alleles with point mutations might confer on clinical isolates. We found that *covS* knockout mutants (Δ*covS* strains) had an impaired growth ability in a normal atmosphere in Todd Hewitt broth compared with parental isolates having wild-type or point-mutated *covS*.

**Conclusions:**

The loss of CovS proteins in *S. pyogenes* may confer greater virulence, but bacteria may also lose the ability to respond to certain external signals recognized by CovS. Therefore, point mutations that retain the function of CovS and confer hypervirulence may have natural selective advantages.

## Background

*Streptococcus pyogenes* is a Gram-positive bacterium that infects the upper respiratory tract, including the tonsils and pharynx, which is responsible for post-infection diseases such as rheumatic fever and glomerulonephritis. *S. pyogenes* also causes severe invasive diseases including necrotizing fasciitis [1-5].

*S. pyogenes* is exclusively a human pathogen and it possesses many virulence factors that help it to resist host defense systems. The production of these factors is thought to be precisely regulated in response to host environmental conditions such as different infection sites or host immune system induction levels [6-8]. In prokaryotes, the regulation of protein production in response to fluctuating environmental conditions depends primarily on two-component regulatory systems, which consist of a sensor histidine kinase and its cognate response regulator [9]. Thirteen two-component regulatory systems have been described in *S. pyogenes*, of which the CovRS system (also known as the CsrRS system) mediates the control of several virulence factors [10-15]. Specific isolates from mice infected with *S. pyogenes* exhibited enhanced virulence in mice owing to spontaneous *covR* or *covS* mutations [10,15,16]. In addition, *covS* mutations were detected more frequently in a panel of clinical isolates from severe invasive streptococcal infections than in a panel of clinical isolates from non-streptococcal toxic shock syndrome [10,16-18]. Thus, Ikebe *et al*. [18] suggested that *covS* mutations are closely associated with the onset of streptococcal toxic shock syndrome.

The strains used for experimental murine infections [10,15,16] and clinical isolates [18] frequently have the M1 serotype, which is the most widely disseminated global serotype [19-21]. Engleberg *et al.*[15] showed that most *covS* mutations were frameshift or nonsense mutations in isolates from mice infected with the M1 strain. In contrast, all of the spontaneous changes in CovS detected in clinical M1 isolates [22] resulted from single amino acid substitutions. Thus, we were interested in why this difference occurred and we hypothesized that it was related to the use of animal-passaged isolates in the first study whereas the latter used clinical isolates. Several *covS* mutations have been reported in other studies [10,16,18] in addition to the two mentioned previously [15,22]. In the current study, we first reviewed the different types of *covS* mutations. This suggested that most of the spontaneous changes in CovS detected in clinical M1 isolates resulted from single amino acid substitutions, whereas most of the *covS* mutations detected in animal-passaged isolates were frameshift mutations. We also showed that *covS* mutations comprising single amino acid substitutions in the clinical isolates partially, but not completely, impaired the functions of CovS. Finally, we present some new findings and discuss why *covS* mutations in clinical isolates are preferentially single amino acid substitutions, whereas animal-passaged isolates tend to have frameshift mutations.

## Results and discussion

### Classification of *covS* mutations

**Figure 1:**
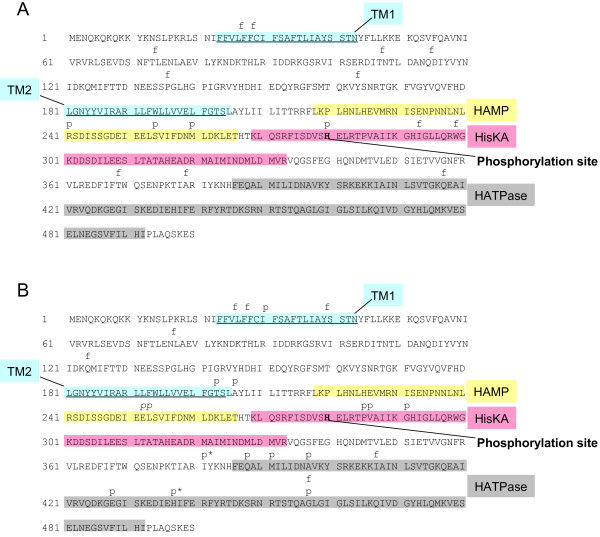
**Summary of the analysis presented in Additional file **1**: Table S1 and Additional file **2**: Table S2. **The various point mutations and frame-shifts indicated were mapped as (p) and (f), respectively, onto the CovS amino acid sequence with putative conserved and functionally-important domains: TM1 and TM2, transmembrane helixes 1 and 2; HisKA, histidine kinase domain (phosphoacceptor); HATPase, histidine-kinase like ATPase. See reference [16] and http://www.ncbi.nlm.nih.gov/protein/7259792?report=genbank&log$=protalign&blast_rank=2&RID=HPZN9V5J01R for additional information about the putative domains. (**A**) The map was made from the Additional file 1: Table S1. A mutation occurred in a functionally-critical phosphorylation site (shown in bold in the CovS amino acid sequence) of the protein in isolates 5448-APD1, 2, 3, 4, 5, and 10. (**B**) The map was made from the Additional file 2: Table S2. Isolate FI01 had two point mutations, which were mapped as (p*), as well as isolate NIH286. Isolate NIH44 had two point mutations, which were mapped as (*p*). The mutation of isolate MGAS2217 was not mapped, because there was not any information about the site.

We investigated all previously reported *covS* mutations. The *covS* mutations were found most frequently in M1 isolates rather than any other serotypes [10,15,18,22], and our unpublished data]. The *covS* mutations detected in M1 strains were divided into two groups: (i) frameshift or nonsense mutations that caused a deletion in functional regions and (ii) point mutations that caused single (or double) amino acid(s) substitutions. Of the 34 *covS* mutations 25 were detected in isolates from mice infected with the M1 strain were frameshift mutations (Figure 1A and Additional file 1). In contrast, 16 of 29 *covS* mutations detected in a panel of clinical isolates comprised single (or double) amino acid(s) substitutions (Figure 1B and Additional file 2). Thus, significantly more frameshift mutations were detected in mouse-passaged isolates, whereas point mutations were most frequent in clinical isolates (*P* < 0.05, Fisher’s exact test).

### Assessment of the function of CovS with an amino acid substitution using two-dimensional gel electrophoresis (2-DE)

**Figure 2:**
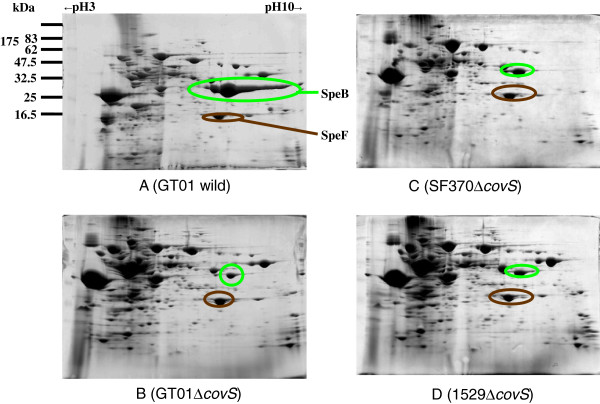
**Two-dimensional gel electrophoresis (2-DE) analysis of exoproteins from *S. pyogenes *GT01 (A), GT01*ΔcovS *(B), SF370*ΔcovS *(C), and 1529*ΔcovS *(D).***S. pyogenes *strains were cultured in 25 ml BHI-Y broth and the proteins in the culture supernatants were subjected to 2-DE analysis using 13 cm Immobiline Drystrip gels (pH 3–10; GE Healthcare Biosciences Co.). Some protein spots identified by LC-MS/MS analysis are shown. The SpeB spot sizes produced from GT01*ΔcovS *(**B**), SF370*ΔcovS *(**C**), and 1529*ΔcovS* (**D**) were smaller than those from GT01 (**A**).

Theoretically, it is possible that a single amino acid substitution has no effect on CovS function, whereas a large deletion may affect domains that are critical for its function. However, we previously observed that all four clinical isolates (strains GT01, K2, AP04, and AP06) with *covS* alleles comprising single amino acid substitutions had lower SpeB production than a clinical isolate having the wild-type *covS* when the culture supernatant proteins were analyzed by 2-DE [22]. It is known that *covS* positively regulates *speB* expression [11,13,14], which suggests that mutated *covS* alleles degrade the function of CovS [22]. Thus, we were interested in how the functions of CovS proteins with single amino acid substitutions were degraded in clinical isolates. Thus, we deleted the *covS*_*GT01*_ allele encoding CovS_GT01_A206S (a substitution of Ala206 with Ser) from the GT01 isolate. As shown in Figure 2, the resulting GT01*ΔcovS* had lower SpeB production than the parental GT01 isolate, suggesting that CovS_GT01_A206S did not completely lose its function.

### Evaluation of the function of CovS with an amino acid substitution based on its NADase activity

*S. pyogenes* secretes NAD^+^-glycohydrolase (NADase) as one of its virulence factors. According to a previous study [10], mouse-passaged derivatives of a strain carrying wild-type CovS exhibited high levels of NADase activity. A comparison of the entire genome of these strains also showed that one of the mouse-passaged derivatives had only one genetic change relative to the parental strain carrying the wild-type CovS, which consisted of a 7-bp insertion in *covS*. Therefore, we were interested in the NADase activity of clinical isolates with *covS* alleles containing single amino acid substitutions. Table 1 shows that strain 1529*ΔcovS* had a NADase activity level of 93.5 U, which was higher than the level of 3.4 U in parental isolate 1529 with wild-type *covS* reported in our previous study [23]. In addition, the activity level of strain 1529*ΔcovS* (93.5 U) was higher than the levels of 62.9 U, 57.0 U, 59.8 U, 60.5 U, and 59.4 U found in the clinical isolates K2, GT01, AP04, AP06, and FI01, respectively, which had point-mutated *covS* alleles, in this study and a previous study [23], although isolate CR01 had a level of 114.3 U. The level of GT01*ΔcovS* was 105.0 U, which was higher than that of parental strain GT01 that carried CovS_GT01_A206S. Thus, the NADase activity levels in isolates with point-mutated *covS* alleles were between those of isolates with wild-type *covS* and isolates with a complete deletion of *covS*. This was consistent with previous reports [22,23], where the levels of NADase in isolates KN01, MDYK, and MUY with wild type *covS* were 6.2 U, 3.0 U, and 3.0 U, respectively.

**Table 1:** **NADase activity of *S. pyogenes *strains**

**Strain**	***covS***	***covR***	**NADase (U**^**a**^**)**	**Reference**
1529	wt	wt	3.4 ± 0.7	[23]
1529*ΔcovS*	*ΔcovS*	wt	93.5 ± 3.5	this study
K2	I30L	wt	62.9 ± 4.6	this study
GT01	A206S	wt	57.0 ± 3.6	[23]
CR01	M391R	wt	114.3 ± 8.7	[23]
AP04	E428G	wt	59.8 ± 2.6	this study
AP06	E428G	wt	60.5 ± 5.4	this study
FI01	I381T+H437R	wt	59.4 ± 4.8	[23]
1529*ΔcovR*	wt	*ΔcovR*	106.7 ± 3.7	this study
GT01*ΔcovS*	*ΔcovS*	wt	105.0 ± 3.2	this study
GT01*ΔcovR*	wt	*ΔcovR*	103.5 ± 6.7	this study
1529*ΔcovS *(pLZ12-km2)	*ΔcovS*	wt	201.9 ± 2.8	this study
1529*ΔcovS *(pLZ-covS_1529_)	wt	wt	130.4 ± 3.4	this study
1529*ΔcovS *(pLZ-covS_1529_I381L)	I381T	wt	176.7 ± 8.9	this study
1529*ΔcovS *(pLZ-covS_1529 _H437R)	H437R	wt	114.5 ± 6.8	this study
1529*ΔcovS *(pLZ-covS_1529_I30L)	I30L	wt	162.7 ± 11.0	this study
1529*ΔcovS *(pLZ-covS_1529_E428G)	E428G	wt	184.8 ± 6.9	this study
1529*ΔcovS *(pLZ-covS_1529_A206S)	A206S	wt	186.7 ± 4.2	this study
1529 (pLZ12-km2)	wt	wt	2.4 ± 0.16	this study

^a ^NADase activity (Units) ± standard error are indicated. One unit of NADase activity is defined as the amount (μg) of β-NAD cleaved per hour per μl culture supernatant, as described previously [23,31].

Next, we attempted to complement 1529*ΔcovS* with wild-type *covS*_*1529*_ or derivatives, which were cloned into plasmid vector pLZ12-Km2. Wild-type *covS*_*1529*_ from isolate 1529 was cloned into pLZ-covS_1529_ and it reduced the NADase activity by 71.5 U from 201.9 U of 1529*ΔcovS* (pLZ12-Km2: control vector) to 130.4 U of 1529*ΔcovS* (pLZ-covS_1529_). In contrast, the NADase activity levels in pLZ-covS_1529_I30L, pLZ-covS_1529_E428G, and pLZ-covS_1529_A206S encoding mutated *covS* alleles from isolates K2, AP04 (or AP06), and GT01 were reduced by 39.2 U, 17.1 U, and 15.2 U, respectively (Table 1 and Figure 2). Thus, the pLZ-covS_1529_I30L, the pLZ-covS_1529_E428G, and the pLZ-covS_1529_A206S certainly retained their abilities to reduce NADase activity, but the abilities were lower than that of the pLZ-covS_1529_ with wild-type *covS.*

These results suggest that amino acid substitutions, such as I30L, E428G, and A206, partially impaired the function of CovS.

### Benefits of partially impaired CovS

**Figure 3:**
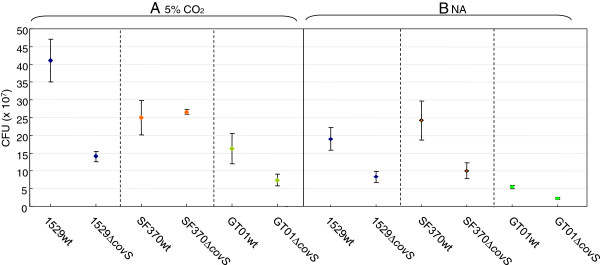
**Growth ability of *covS *mutant *S. pyogenes *in THY broth. **The CFU/ml after 23 h, THY broth culture of isogenic *covS *mutant strains (1529*ΔcovS, *SF370*ΔcovS, *and GT01*ΔcovS*)*, *and parental isolates (1529, SF370, and GT01) are shown. At least three independent experiments were performed and they always yielded essentially the same results. The error bars indicate the standard errors of the means. (**A**) 5% CO2 was used as an experimental condition. The CFU/ml for overnight THY broth cultures of strains 1529*ΔcovS *and GT01*ΔcovS *were lower than those for their parental strains 1529 and GT01, respectively. The CFU/ml for overnight THY broth cultures of SF370*ΔcovS *were similar to that for the parental strain SF370. (**B**) Natural atmosphere (NA) was used as an experimental condition.

**Figure 4:**
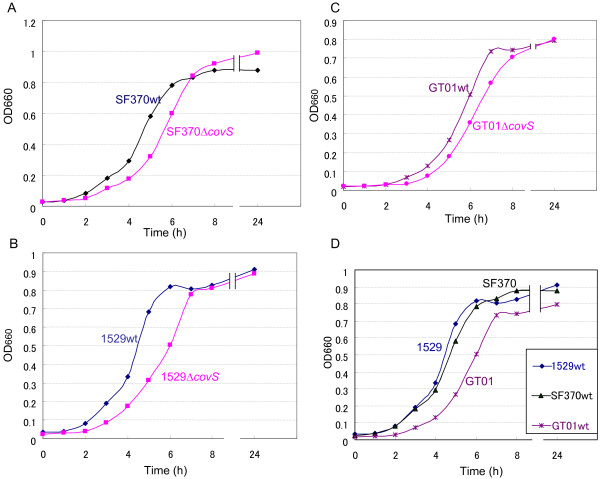
**Growth curves of *covS *null mutants in THY broth. **Three independent experiments produced essentially the same results. Representative data from three independent experiments are shown. (**A-C**) SF370*ΔcovS*, 1529*ΔcovS*, and GT01*ΔcovS* all exhibited delayed growth compared with that of their parental strains SF370, 1529, and GT01, respectively. (**D**) GT01 exhibited delayed growth compared with strains 1529 and SF370.

According to previous studies [10,16-18], CovS negatively regulates the expression of certain virulent genes; therefore, a mutation in *covS* may increase the virulence in mouse models of infection where it plays a crucial role in the onset of severe invasive infections. However, the loss of CovS function means that *S. pyogenes* can no longer adjust to environmental fluctuations. For example, environmental Mg^2+^ is thought to be recognized by the CovS sensor protein [24]. Therefore, the partial loss of CovS function may be favorable in nature, but not under laboratory conditions. This hypothesis led us to investigate the benefits of *covS* in *S. pyogenes*. Previously, Trevino *et al.*[25] showed that a *covS* mutated strain had a lower growth ability than the parental wild-type strain in human saliva, but not in Todd Hewitt broth, which is the standard broth used to culture *S. pyogenes*. We were interested in the factor present in human saliva that is recognized by CovS; therefore, we repeated this experiment using the isolates 1529, SF370, and GT01. Bacteria were cultured under essentially the same conditions as those described previously [25]. However, the CFU (colony forming units)/ml for overnight THY broth cultures of strains 1529*ΔcovS,* SF370*ΔcovS,* and GT01*ΔcovS* were lower than or similar to those of their parental strains; i.e., 1529, SF370, and GT01, respectively (Figure 3A), whereas the CFU/ml for overnight THY broth cultures of the isogenic mutant 2221covS::7 bp was four times that of the parental strain MGAS2221 reported in the previous study [25]. Thus, we observed two discrepancies: (i) between our results using isolates 1529, SF370, and GT01 (Figure 3A), and the previous results based on isolate MGAS2221 [25] and (ii) between our results with isolate 1529 (or GT01) and SF370. These discrepancies may be because of strain specificities. We did not have strain MGAS2221, so we further investigated the discrepancy between strains SF370 and GT01 or 1529. First, we analyzed the growth curves of the *covS* mutated strains. SF370*ΔcovS,* 1529*ΔcovS,* and GT01*ΔcovS* all showed delayed growth compared with that of their parental strains; i.e., SF370, 1529, and GT01, respectively (Figures 4A–C). Thus, there was no discrepancy between strains SF370, GT01, and 1529 in terms of their growth kinetics. In addition, GT01 exhibited delayed and advanced growth compared with strain 1529 (or SF370) and strain GT01*ΔcovS* (Figures 4D and C), which was consistent with our hypothesis that the A206S substitutions partially impaired the function of CovS.

**Figure 5:**
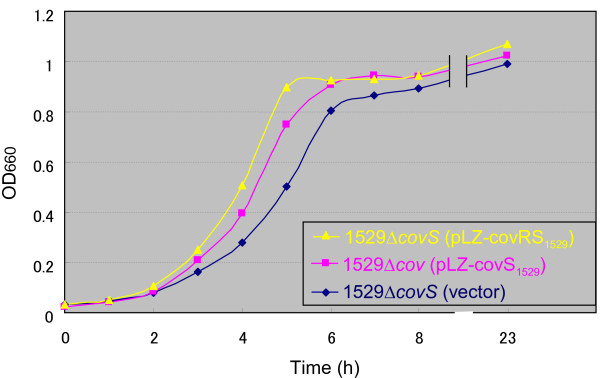
**Growth curves of streptococcal strains in THY broth. **Bacteria were cultured in THY broth supplemented with kanamycin (62.5 μg/ml) and the experiments were performed as described in Figure 4.

**Figure 6:**
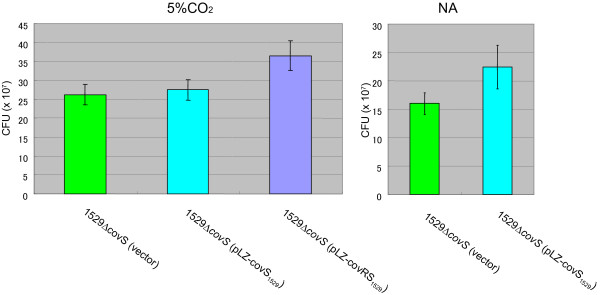
**Growth of *covS *mutant *S. pyogenes *in THY broth with 5% CO**_**2 **_**or NA. **These experiments were performed as described in Figures 3. The error bars indicate the standard errors of the means.

**Figure 7:**
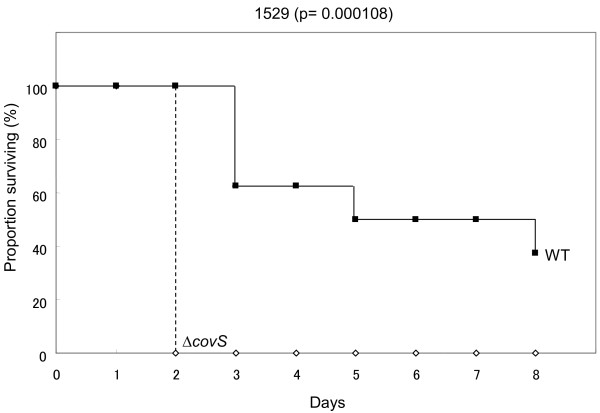
**Survival after skin inoculation with *S. pyogenes *strain 1529 or isogenic mutant 1529*ΔcovS. ***Data were from two independent experiments and a total of eight mice for each challenged strain. *P *< 0.01 for comparison between strains.

The growth abilities of SF370 and its isogenic *covS* mutant SF370*ΔcovS* in THY broth differed from each other when evaluated on the basis of, but not the CFU/ml in overnight cultures (Figure 3A), the growth curves (Figure 4A). This new discrepancy may have occurred because the overnight culture, but not the growth curve, was conducted in 5% CO_2_, which was the condition described in a previous study [25]. Therefore, we prepared overnight cultures of wild-type SF370 (SF370wt) and SF370*ΔcovS* in natural atmosphere (NA) conditions. As shown in Figure 3B, the CFU/ml for SF370*ΔcovS* was lower than that of its parental strain SF370. Finally, we performed supplementary and supporting experiments to test the reliability of this study. *covS* and *covRS* cloned into a plasmid vector complemented the delayed growth of 1529*ΔcovS* (Figure 5). pLZ-covS_1529_ and pLZ-covRS_1529_ increased the CFU/ml for overnight THY broth cultures of strain 1529*ΔcovS* (Figure 6). As shown in Figure 7, 1529*ΔcovS* was hypervirulent in a mouse infection model compared with the parental strain 1529 (P<0.01), as shown with other strains and their isogenic *ΔcovS* mutants in previous studies [10,16].

## Conclusions

On the basis of our results and those from previous studies we concluded that the loss of *covS* increases the virulence of *S. pyogenes* (which is advantageous *in vivo*). However, the loss of *covS* also impaired the growth ability of this organism in THY broth (which is disadvantageous *in vitro*). Therefore, the CovRS system may confer benefits in stages when virulent gene expression is not required. The expression of many genes is precisely regulated so they are expressed only when required; e.g., catabolite repression. Therefore, partial attenuation of the CovRS system to promote resistance to the host defense system appears to be a wise choice for survival in nature.

We did not determine the components recognized by the CovS sensor proteins in our experimental conditions; i.e., THY broth, natural atmosphere, or 5% CO_2_. However, it was probably not the Mg^2+^ ion, which was suggested previously [24], because we did not add Mg^2+^ ion to THY broth. Therefore, we propose that CovS can sense other signals in addition to the Mg^2+^ ion.

## Methods

### Bacterial strains

Streptococcal strains were isolated as the causative organisms in patients from Japan [22,23]. *S. pyogenes* (GAS) strain SF370, which was the most prevalent database reference isolate (accession number NC_002737), was provided by J. J. Ferretti [26,27]. Streptococcal strains were cultured in brain–heart infusion (E-MC62, EIKEN Chemical Co., Tokyo, Japan) supplemented with 0.3% yeast extract (BD, Sparks, MD, USA), (BHI-Y) broth or Todd Hewitt broth (BD, Sparks, MD, USA) supplemented with 0.2% yeast extract broth (THY) unless otherwise stated.

### Production of *covS* knockout strains

We constructed *S. pyogenes* strain 1529*ΔcovS* as described previously [28]. Strains GT01*ΔcovS* and SF370*ΔcovS* were constructed using the same strategy [28].

### Two-dimensional gel electrophoresis (2-DE)

Each bacterial isolate was cultured in BHI-Y at 37°C overnight without agitation. Exoproteins from the culture supernatant were prepared as described previously [22]. In brief, all sample pellets derived from bacterial culture supernatant were dissolved in dehydration solution, which consisted of 7.8 M urea, 2 M thiourea, 2% CHAPS, 0.6% dithiothreitol, and 0.5% IPG buffer. The samples were loaded onto 13 cm Immobiline DryStrip gels (pH 3–10, GE Healthcare Biosciences Co. Piscataway, NJ, USA). The first-dimensional electrophoresis conditions were carried out according to the manufacture’s instruction. Second-dimensional SDS-PAGE separation was performed as described previously [22]. The experiments were repeated at least 3 times to confirm their reproducibility.

### Production of *covR* knockout strains

To construct the plasmid for the *covR* knockout mutant, the 5^′^ end of *covR* (fragment 1) was amplified using the oligonucleotide primer *cov*R-n6 (5^′^-GGCTAGCCTTTAGAGAATATGGTTACT-3^′^) with an *Nhe*I restriction site and primer *cov*R-c2 (5^′^-TCCCCCGGGCTTTGTCATTTATACCAACC-3^′^) with an *Sma*I restriction site, while the 3^′^ end of *covR* (fragment 2) was amplified using the primer *cov*R-n7 (5^′^-TCCCCCGGGGAGAAATAAGTCATATGGAA-3^′^) with an *Sma*I restriction site and primer *cov*S-c10 (5^′^-GGACTAGTATGTAAAATTAGAGTCCACC-3^′^) with an *Spe*I restriction site. Fragment 2 was digested with *Sma*I and *Spe*I before its insertion into multicloning site 2 in the plasmid pFW12 [29]. The resulting plasmid was digested using *Nhe*I and *Sma*I, and the spc1 DNA fragment containing *aad9* (promoterless spectinomycin resistance gene), which was obtained from a *Sma*I-digested fragment of pSL60-1 [29], and the *Nhe*I-*Sma*I-digested fragment 1 were inserted. This plasmid, *covR*::*aad9*/pFW12, was a suicide vector for *S. pyogenes*. To prepare competent cells, strains 1529 and GT01 were harvested in the early to mid-log phase (OD_660_, 0.4) and washed twice with 0.5 M sucrose buffer. The suicide vector construct, *covR*::*aad9*/pFW12, was transformed into strains 1529 and GT01 via electroporation. The conditions for electroporation were 1.25 kV/mm, 25-μF capacitance, and 200-Ω resistance, and it was performed using a GenePulser II instrument (Bio-Rad, Hercules, CA). After incubation at 37°C for 3 h, competent cells were spread onto BHI agar plates containing 0.3% yeast extract and spectinomycin (final concentration, 100 μg/ml). Selected colonies were cultured from the plates. The cultured bacteria were washed once with saline, resuspended in 10 mM Tris-1 mM EDTA, and boiled for 10 min. Genomic DNA was obtained from the supernatant of the boiled bacteria. The double-crossover replacement was analyzed by PCR using genomic DNA. Successful double-crossover replacement was further confirmed by DNA sequencing.

### Quantification of the NADase activity in the bacterial supernatant

NADase activity was determined using the method of Stevens *et al*. [30] as described previously [31].

### Plasmids

pLZ-covS_1529_, pLZ-covS_1529_I30L, and pLZ-covS_1529_E428G were constructed as described previously [22]. To construct pLZ-covS_1529_A206S, the DNA fragment was amplified using the oligonucleotide primers covR-n2 (5^′^-CTTTAGAGAATATGGTTACT-3^′^), covS-c2 (5^′^-GTAATTACATTTTGGACAAC-3^′^), and GT01 genomic DNA as templates with *TaKaRa Ex Taq* DNA polymerase (Takara, Ohtsu, Japan). The fragment consisted of *covR*_*GT01*_, *covS*_*GT01*_*,* and their 5^′^-noncoding region, which possibly contained the promoter region. This fragment was cloned into the pGEM-T vector (Promega, Madison, WI, USA). The resultant plasmid was digested with *Eco*RI and ligated into the same site in the pLZ12-Km2 plasmid [32] (pLZ-covRS_GT01_). To construct a plasmid containing only the *covS*_*GT01*_ region, inverse PCR was conducted using two primers, covR-c2Sma (5^′^-TCCCCCGGGCTTTGTCATTTATACCAACC-3^′^) and covR-n7Sma (5^′^-TCCCCCGGGGAGAAATAAGTCATATGGAA-3^′^), with pLZ-covRS_GT01_ plasmid DNA as template and Prime-STAR HS DNA polymerase (Takara) to eliminate the *covR* region. This blunt-ended PCR product was treated with T4 polynucleotide kinase (Takara) and self-ligated. The resultant plasmid was pLZ-covS_1529_A206S. pLZ-covRS_1529_ encoding the *covRS*_*1529*_ operon of isolate 1529 was constructed as described previously [22]. All of the *covRS* DNA sequences were confirmed by sequencing.

### Mouse model of invasive skin tissue infection

All animal studies conducted comply with federal and institutional (the Committee on the Ethics of Animal Experiments of the Nagoya City University) guidelines. The protocol was approved by the Committee on the Ethics of Animal Experiments of the Nagoya City University (Permit Number: H23M-07). All efforts were made to minimize suffering.

The ability of *S. pyogenes* to cause local skin lesions and necrosis in mice after skin inoculation was assessed using a similar procedure to that described previously [23,33]. Three-week-old female ICR mice (10–12 g) were anesthetized with sevoflurane and the skin of the left flank was laid bare by separating the hair with an alcohol swab, unless indicated otherwise. Bacteria (0.2 ml; 2 × 10^7^ CFU/mouse) grown in BHI-Y were injected immediately beneath the surface of the skin using a 27-gauge needle so a superficial bleb appeared below the skin surface. The number of CFU injected was verified in each experiment by plating bacteria on BHI-Y or sheep blood agar plates and counting the CFU.

### Statistical analysis

The survival times were assessed using a log-rank comparison. The R program was used for the statistical analysis http://bioinf.wehi.edu.au/software/russell/logrank/*webcite*. *P* ≤ 0.05 was considered significant.

## Availability of supporting data

There are two supplementary tables.

## Abbreviations

NADase: NAD^+^-glycohydrolase; NA: Natural atmosphere; THY: Todd Hewitt yeast.

## Competing interests

There are no competing interests.

## Authors’ contributions

IT conceived the study. IT, RO, and TH designed and performed the experimental work with help by YZ and MI. All authors contributed to the data analysis. IT wrote the original manuscript. TH helped to produce the final manuscript. All authors approved the final manuscript.

## Supplementary Material

Additional file 1: Table S1 *csrS *mutations from mouse-passaged isolates of M1 *S. pyogenes.* Click here for file

Additional file 2: Table S2 *csrS *mutations from human clinical isolates of M1 *S. pyogenes.* Click here for file
