# Supplementary material for: Partial loss of CovS function in Streptococcus pyogenes causes severe invasive disease
Source: BMC Res Notes. 2013 Mar 28;6:126. doi: 10.1186/1756-0500-6-126 (PMC3637574; doi:10.1186/1756-0500-6-126)
Supplement: Additional file 1: Table S1 — csrS mutations from mouse-passaged isolates of M1 S. pyogenes. [file 1756-0500-6-126-S1.doc]

**Additional Table 1. *covS* mutations from mouse-passaged isolates of M1 *S. pyogenes.***

Type of mutation or consequence of the mutation Isolate name reference

**(Small changes in the deduced amino acid sequence: aa substitution)**

Met→Thr at aa 260 UMAA2752 [15]

Ser→Phe at aa 254 UMAA2880 [15]

Arg→Ser at aa 241 UMAA2887 [15] His→Tyr at aa 280 5448-APD1 [16]

5448-APD2 [16]

5448-APD3 [16]

5448-APD4 [16]

5448-APD5 [16]

5448-APD10 [16]

**(Large deletion in the deduced amino acid sequence)**

Frameshift (Δ nt 76 to 83) Early termination after aa 35 UMAA2207 [15]

UMAA2211 [15]

Frameshift (Δ nt 492) Early termination after aa 181 UMAA2209 [15]

IS1548 insertion at nt 342 Insertional inactivation UMAA2210 [15]

2 bp deletion at nt 321 Early termination UMAA2759 [15]

5 bp deletion at nt 218 Early termination UMAA2762 [15]

2 bp insert at nt 1136 Early termination UMAA2882 [15]

1 bp insert at nt 1105 Early termination UMAA2857 [15]

UMAA2877 [15]

UMAA2878 [15]

UMAA2881 [15]

UMAA2884 [15]

Δnt 1250 Early termination 21PL1 [10]

21PL3 [10]

24SS1 [10]

24SS2 [10]

24SS3 [10]

24SL2 [10]

7 bp insertion 202 amino acid truncation of CovS 26PL1 [10]

26PL2 [10]

26PL3 [10]

Δ nt 83 Truncation 5448-APD6 [16]

5448-APD7 [16]

1 bp insertion at nt 877 Termination at aa 300 5448AP [16]

Δ nt 406–1503 Truncation 5448-APD9 [16]
