# Supplementary material for: Partial loss of CovS function in Streptococcus pyogenes causes severe invasive disease
Source: BMC Res Notes. 2013 Mar 28;6:126. doi: 10.1186/1756-0500-6-126 (PMC3637574; doi:10.1186/1756-0500-6-126)
Supplement: Additional file 2: Table S2 — csrS mutations from human clinical isolates of M1 S. pyogenes. [file 1756-0500-6-126-S2.doc]

**Additional Table 2. *covS* mutations in human clinical isolates of M1 *S. pyogenes.***

Type of mutation or consequence of the mutation Isolate name reference

**(Small changes in the deduced amino acid sequence: aa substitution)**

Ala→Val at aa 397 MGAS6184 [10]

Val→Phe at aa 286 MGAS294 [10]

Ile → Leu at aa 30 K2 [22]

Glu→Gly at aa 428 AP06 [22]

AP04 [22]

Ile→Thr at aa 381, and His→Arg at aa 437 FI01 [22]

Ala→Ser at aa 206 GT01 [22]

NIH222 [18]

Met→Arg at aa 391 CR01 this study

Delete Glu at aa 252, and Leu→Val at aa 253 NIH44 [18]

Gln→Arg at aa 388 NIH205 [18]

Gly→Val at aa 457 NIH220-1 [18]

Gly→Arg at aa 291 NIH243-1 [18]

Ile→Tyr at aa 381 and His→Arg at aa 437 NIH286 [18]

Thr→Lys at aa 203 NCU183 this study

Pro→Ser at aa 285 NCU358 this study

**(Large deletion in the deduced amino acid sequence)**

Δnt 80 MGAS5005 [10]

1 bp insertion MGAS2217 [10]

5 bp deletion stop at aa 407 NIH73 [18]

NIH83 [18]

1 bp deletion stop at aa 76 NIH102 [18]

1 bp deletion stop at aa 35 NIH202-2 [18]

NIH213-3 [18]

NIH235 [18]

NIH397 [18]

NIH366* [18]

1 bp deletion stop at aa 457 NIH253-1 [18]

11 bp deletion stop at aa 39 NIH314 [18]

CAG→TAG at codon 124 Gln→stop at aa 124 11I-5 this study

The Isolates which have mutations in both *covS* and *covR* were not included.

*, NIH366 also had another mutation (Rgg, delete from aa129 to 247).
